# Editorial: Impact of genome instability on human health

**DOI:** 10.3389/fmolb.2023.1243968

**Published:** 2023-07-18

**Authors:** Swagat Ray, Sanjiban Chakrabarty, Arnab Ray Chaudhuri

**Affiliations:** ^1^ Department of Life Sciences, School of Life and Environmental Sciences, University of Lincoln, Lincoln, United Kingdom; ^2^ Department of Cell and Molecular Biology, Manipal School of Life Sciences, Manipal Academy of Higher Education, Manipal, Karnataka, India; ^3^ Center for DNA Repair and Genome Stability (CDRGS), Manipal Academy of Higher Education, Manipal, Karnataka, India; ^4^ Department of Molecular Genetics, Erasmus MC Cancer Institute, Erasmus University Medical Center, Rotterdam, Netherlands

**Keywords:** DNA damage, PARP inhibition (PARPi), genomic instability, cancer, transcription factor

Genomic instability, characterized by alterations in DNA structure and function, has emerged as a crucial factor influencing human health and disease. In recent years, a growing body of research has focused on unraveling the intricate relationship between genomic instability and its impact on various conditions such as cancer, aging, and genetic disorders, which are reviewed here (Abugable et al., 2019; Tiwari and Wilson, 2019; Nelson and Dizdaroglu, 2020; Yousefzadeh et al., 2021). This editorial presents a synthesis of four recently published articles in Frontiers in Molecular Biosciences that delve into the mechanisms and consequences of genomic instability in different contexts. These studies underscore the role of homologous recombination deficiency in epithelial ovarian cancer, the modulation of a transcription factor in the pathogenesis of breast cancer, the epigenetic landscape of Klinefelter syndrome, and the immunomodulatory effect of PARP inhibitors (PARPi) against cancer. Together, these findings contribute to our understanding of genomic instability and pave the way for improved diagnostics, targeted therapies, and interventions to improve human health.

Yi et al. contribute to the understanding of genomic instability and its impact on human health, specifically in the context of epithelial ovarian cancer (EOC). Their study investigates the association between the detection of homologous recombination deficiency (HRD) and postoperative survival in EOC patients. Their findings shed light on the prevalence of HRD in EOC, with a substantial proportion (72.4%) of tumors exhibiting HRD scores of ≥42. Additionally, BRCA mutations were identified in a subset of patients. These results emphasize the role of genomic instability, particularly HRD, in the development and progression of EOC. While the study did not establish a significant association between HRD scores and specific homologous recombination repair (HRR) genes, it did identify a positive correlation between HRD scores and chromosomal instability (CIN). This association underscores the intricate relationship between genomic instability and HRD, suggesting that mechanisms influencing chromosomal stability play a crucial role in the development of HRD. Importantly, the study demonstrated that an HRD score of >23 was associated with better postoperative progression-free survival (pPFS). This finding implies that the assessment of the HRD score may serve as a prognostic tool for EOC patients, providing valuable information for treatment decisions and potentially guiding the use of poly (ADP-ribose) polymerase inhibitor (PARPi) therapy. Nonetheless, additional validation in a larger patient cohort is necessary to confirm these findings, as the study was conducted with a limited number of participants.

In their research, Kuthethur et al. explore the effects of modulating a specific transcription factor on the pathogenesis of breast cancer. They study the role of FOXM1, an oncogenic transcription factor, in cell proliferation, DNA damage response, and chemoresistance in cancer cells. The study reveals that miR-4521, a specific microRNA, directly targets and reduces the expression of FOXM1 in cancer cells. This downregulation of FOXM1 leads to increased levels of reactive oxygen species (ROS) and DNA damage within the cancer cells. Moreover, miR-4521-mediated downregulation of FOXM1 inhibits cell proliferation, invasion, cell cycle progression, and the process of epithelial-to-mesenchymal transition (EMT) in breast cancer. These findings highlight the potential of miR-4521 as a promising therapeutic approach for the treatment of breast cancer.

Klinefelter syndrome (KS), characterized by the presence of an additional X chromosome in men (47XXY), is associated with altered gene expression and abnormal physical traits. Miao et al. explore the epigenetic landscape of 47XXY individuals using state-of-the-art sequencing technologies, shedding light on the underlying mechanisms and potential implications for diagnosis and therapy. By examining DNA methylation patterns in plasma samples from adult 47XXY individuals and chromatin accessibility in fetal amniotic cells, the researchers observed abnormal DNA methylation throughout the genome, particularly in the X chromosome. Notably, amniotic cells from 47XXY fetuses exhibited dynamic and increased chromatin accessibility compared to their typical male and female counterparts (46XX and 46XY). The researchers hypothesized that the abnormal opening of chromatin during early developmental stages may trigger the observed whole-genome methylation changes in 47XXY. The study used two sequencing methods, Whole-Genome Bisulfite Sequencing (WGBS) (Lister et al., 2009) and Assay for Transposase-Accessible Chromatin Using Sequencing (ATAC-seq) (Buenrostro et al., 2015), to construct the comprehensive epigenetic landscape of 47XXY.

The final work in this Research Topic is a comprehensive investigation into the potential of PARP inhibitors to enhance the immune response against cancer. DNA damage response (DDR) deficiencies contribute to genomic instability, a hallmark of cancer, and PARP enzymes play a vital role in DDR pathways, influencing cell fate following DNA damage (Ray Chaudhuri and Nussenzweig, 2017). PARP_i_ targeting PARP1 and PARP2, key players in DDR, have been approved for the treatment of various tumor types due to their druggable nature. Hunia et al. highlight the multifaceted roles of PARP1/2 in cellular processes and elucidate the mechanism of action of PARPi from laboratory research to clinical application. The authors also provide insights into ongoing clinical trials demonstrating the synergistic effects of PARPi and immune checkpoint inhibitors (ICIs). Diagnostic tools for therapeutic development are introduced, and the prospects and limitations of this approach are discussed.

In conclusion, these recent studies have significantly contributed to our understanding of the role of genome instability in human health, specifically in the context of cancer and genetic disorders. The findings underscore the importance of genomic instability, such as homologous recombination deficiency and altered gene expression, in the development and progression of diseases like epithelial ovarian cancer, breast cancer, and Klinefelter syndrome. Furthermore, the identification of potential prognostic markers and therapeutic targets, such as HRD scores and miR-4521, highlights the clinical implications of these research findings. Moreover, the comprehensive review of PARP inhibitors provides valuable insights into their mechanism of action and potential synergistic effects with immunotherapy. Taken together, these studies pave the way for improved diagnostics, targeted therapies, and potential interventions for individuals affected by these conditions, ultimately advancing the field of genomic medicine.
